# An Attempt to Identify the Medaka Receptor for Somatolactin Alpha Using a Reverse Genetics Approach

**DOI:** 10.3390/genes14040796

**Published:** 2023-03-26

**Authors:** Yuko Moroki, Mamiko Komori, Yuko Ogawa, Erina Nagumo, Haruna Ohno, Shoji Fukamachi

**Affiliations:** Department of Chemical and Biological Sciences, Japan Women’s University, Tokyo 112-8681, Japan

**Keywords:** medaka fish, phylogenetic reconstruction, genome editing, frameshift mutations, growth hormone receptor, somatolactin alpha receptor, body color

## Abstract

Somatolactin alpha (SLα) is a fish-specific hormone involved in body color regulation. The growth hormone (GH) is another hormone that is expressed in all vertebrates and promotes growth. These peptide hormones act by binding to receptors (SLα receptor (SLR) and GH receptor (GHR)); however, the relationships between these ligands and their receptors vary among species. Here, we first performed phylogenetic tree reconstruction by collecting the amino-acid sequences classified as SLR, GHR, or GHR-like from bony fish. Second, we impaired SLR or GHR functions in medaka (*Oryzias sakaizumii*) using CRISPR/Cas9. Lastly, we analyzed SLR and GHR mutants for phenotypes to deduce their functions. Phylogenetic tree reconstruction was performed using a total of 222 amino-acid sequences from 136 species, which revealed that many GHRa and GHRb are vaguely termed as GHR or GHR-like, while showing no orthologous/paralogous relationships. SLR and GHR mutants were successfully established for phenotyping. SLR mutants exhibited premature lethality after hatching, indicating an essential role for SLR in normal growth. GHR mutations did not affect viability, body length, or body color. These results provide no evidence that either SLR or GHR functions as a receptor for SLα; rather, phylogenetically and functionally, they seem to be receptors for GH, although their (subfunctionalized) roles warrant further investigation.

## 1. Introduction

Somatolactin alpha (SLα) is a peptide hormone specific to fish that is secreted from the pars intermedia of the pituitary gland and regulates body color in medaka [1,2,3,4]. The body color of fish is adjusted according to the density and physiological condition of pigment cells, termed chromatophores, located on the body surface [5,6]. Chromatophores are classified into six types, according to the color of the pigment granules they contain: melanophores, erythrophores, xanthophores, leucophores, iridophores, and cyanophores. Medaka have four types of chromatophores and a spontaneous mutant, called *color interfere* (*ci*), which has a brighter gray color on the body surface than its wild-type (WT) counterpart because of an increase in leucophores and iridophores and the decrease in melanophores and xanthophores [2]. Conversely, the excessive expression of SLα in *ci* causes a darker yellow color on the body surface compared to *ci* with reduced expression because of the increase in melanophores and xanthophores and the decrease in leucophores and iridophores [3].

The growth hormone (GH) is another peptide hormone that is expressed in all vertebrates. This hormone is secreted from the anterior pituitary gland and is involved in growth promotion (e.g., somatic growth, metabolism of lipids and carbohydrates, and cell differentiation), reproduction, osmotic regulation, and immunogenicity [7]. The overexpression of GH in medaka (*O. sakaizumii*) triggers an increase in body size and severe infertility in most female fish [8].

SLα and GH are the closest relatives in the GH/prolactin (PRL) family and function by binding to the extracellular domain of their receptors (the SLα receptor (SLR) and GH receptor (GHR)) [9,10]. The amino-acid identity between SLα (231 residues) and GH (210 residues) in medaka is 23% [8]. Hence, we assumed previously that SLR and GHR would also be the closest relatives, which could be a clue for identifying an SLR in fish that is absent in land vertebrates.

Fukada et al. revealed the differences in the GH-binding ability of the two GH receptors present in masu salmon (*Oncorhynchus masou*) using an in vitro approach: one binds to both SLα and GH with low binding specificity, and more strongly to SLα than to GH; the other binds to GH alone [9,10]. Therefore, these receptors were hypothesized to be SLR and GHR, respectively. Conversely, both GHR1 and GHR2 bind to GH, but not to SLα, in zebrafish (*Danio rerio*) [11]. One of the two GHRs (GHR1) also binds specifically to GH in Japanese eels *(Anguilla japonica*) [12]. Therefore, the binding ability strangely varies according to the fish species. In addition, the SLR/GHR nomenclature is currently quite confusing; thus, it is necessary to clarify the evolutionary and functional relationships between these receptors and ligands (see Ocampo Daza et al. [13,14] for details).

Phylogenetic tree construction is a method that has been used to improve the understanding of genetic relationships because it can visualize the clade position of the gene sequences systematically and help elucidate the relationships among genes under an evolutional process. Reverse genetics is another useful technique for understanding gene function by genetically engineering specific nucleic acid sequences to assess phenotypes. To ensure the impairment of receptor function, the Clustered Regularly Interspaced Short Palindromic Repeats (CRISPR)/CRISPR-associated protein 9 (CRISPR/Cas9) system [15,16,17] is a good candidate for generating frameshift mutations on the *SLR* and *GHR* genes and for assessing phenotypes.

Therefore, in this study, first, we performed an in silico phylogenetic tree reconstruction using amino-acid sequences from bony fish classified as SLR, GHR, or GHR-like using the RefSeq database at the National Center for Biotechnology Information (NCBI). Based on the position of the gene sequences, we propose the GHRa/GHRb nomenclature instead of the SLR/GHR one. Second, we impaired the receptor functions of SLR and GHR in medaka, using the CRISPR/Cas9 system, to produce mutants with frameshift mutations and establish various lineages. Lastly, we analyzed the phenotype of the *slr* and *ghr* mutants and estimated the function of these receptors.

## 2. Materials and Methods

### 2.1. Animals

Japanese rice fish of the HNI strain (WT) were used to establish new strains with specific mutations in the *SLR* or *GHR* gene induced by the CRISPR/Cas9 system. All fish were hatched and bred in our laboratory. This study was reviewed and approved by the Animal Experiment Committee of Japan Women’s University. Fertilized medaka eggs were reared in a Petri dish. After hatching, they were reared in a static water tank. A few weeks after hatching, the grown fish were transferred to a circulating tank with filtered water that was maintained at a temperature between 25 °C and 28 °C with light provided by white LED bulbs and white fluorescent lamps under a 14 h/10 h light/dark cycle. Larval fish were fed paramecium or powder food, and adult fish were provided with brine shrimp and commercial flake food (TetraMin) five times a day between 10h00 and 18h00.

### 2.2. Phylogenetic Tree Reconstruction

The amino-acid sequences from bony fish that were classified as SLR, GHR, or GHR-like were collected from the RefSeq database at the NCBI. GHRs from several land vertebrates were also collected for use as an outgroup. The listed sequences were aligned using ClustalW, and many redundant (e.g., isoforms X1, X2, or X3) or apparently odd (e.g., exceptionally short/long) sequences were manually eliminated. For some sequences, we trimmed the N-terminal region in accordance with other sequences. A maximum-likelihood tree was drawn using the RAxML (version 8.10.12)-installed Genetyx-Mac software (version 21.2.0), and some sequences with phylogenetic positions that apparently contradicted the traditional tree (likely because they were not GHR orthologs) were excluded from the list (we suspect that these were genuine SLRs). This final list (Supplementary Table S1) was used for phylogenetic reconstruction using the RAxML-installed Genetyx-Mac software with the amino-acid substitution model of PROTGAMMA, the substitution matrix of BLOSUM62a, and a bootstrapping value of 100.

### 2.3. Preparation and Microinjection of the gRNA and Cas9 mRNA

**Figure 1:**
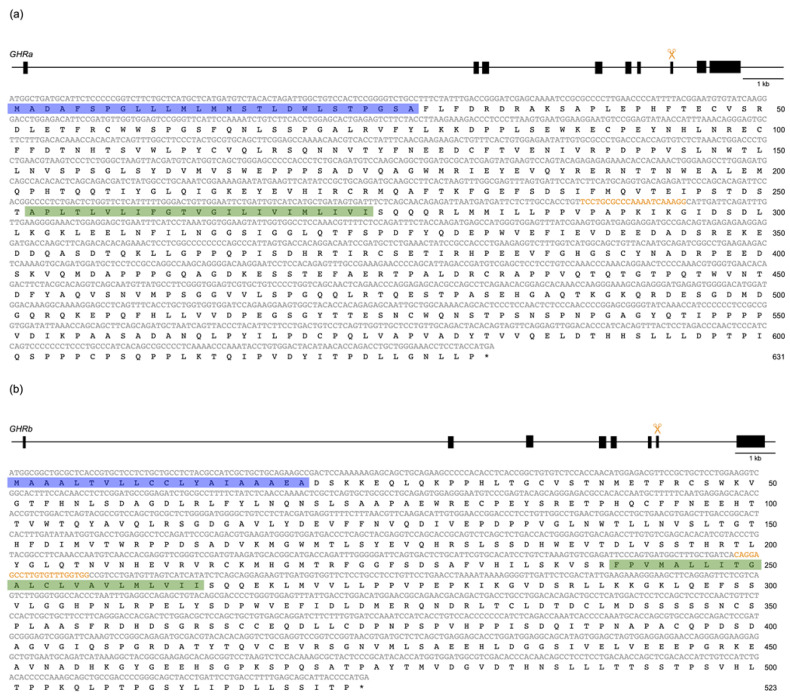
Genomic structure of the *somatolactin alpha receptor* (*SLR: GHRa*) (**a**) and *growth hormone receptor* (*GHR: GHRb*) (**b**) genes (GenBank accession numbers: DQ002886 and DQ010539, respectively). The black boxes represent translated regions (the 5′/3′ UTRs are not shown). The orange scissors indicate the approximate positions of the target sequences in CRISPR/Cas9. The cDNA and amino-acid sequences are shown in gray and black, respectively. The target sequences are shown in orange. The signal peptides predicted by SignalIP 5.0 and the transmembrane regions predicted by TMHMM 2.0 are highlighted in blue and green, respectively.

The target sequences for CRISPR/Cas9 were TCCTGCGCCCAAAATCAAAGG for *SLR* (Figure 1a) and CAGGAGCCTTGTGTTTGGTGG for *GHR* (Figure 1b). Properly designed oligonucleotide DNAs were purchased from Thermo Fisher Scientific (Waltham, MA, USA). A circular pDR274 plasmid (Addgene plasmid 42,250) was linearized with BsaI and confirmed by electrophoresis on a 1% agarose gel with ethidium bromide staining. The linear plasmid was excised from the gel and purified with the Wizard^®^ SV Gel and PCR Clean-UP System (Promega, Madison, WI, USA). The annealed double-stranded oligonucleotides were subcloned into linearized pDR274 using the Ligation high Ver.2 reagent (Toyobo, Osaka, Japan), and proper insertion was confirmed by colony polymerase chain reaction (PCR) using M13 primers and direct sequencing. The double-stranded oligonucleotide-inserted pDR274 plasmid and the hCas9 plasmid were digested with DraI and NotI, respectively. The gRNA and capped *Cas9* mRNA were synthesized using the AmpliScribe T7-flash Transcription Kit (Illumina, San Diego, CA, USA) and the mMESSENGE mMACHINE SP6 kit (Thermo Fisher Scientific), respectively. After DNase treatment, the transcripts were purified using the Rneasy Mini Kit (Qiagen, Venlo, Netherlands) and confirmed by electrophoresis on a 1% agarose gel. The concentration of the gRNA was measured using a NanoDrop Lite instrument (Thermo Fisher Scientific). All procedures were in accordance with the manufacturers’ protocols.

A mixed solution of gRNA and *Cas9* mRNA (final concentrations of 25 ng/µL and 100 ng/µL, respectively) was placed in an injection needle and set in an IM-12 microinjector (Narishige, Tokyo, Japan). The RNA mixture (one type of gRNA and the *Cas9* mRNA when checking mutagenesis efficiency, or two types of gRNA (SLR8 and GHR6) and the *Cas9* mRNA for actual mutagenesis) was injected into the cytoplasm of 1-cell-stage medaka embryos of the HNI strain (WT) under an SMZ18 stereoscopic microscope (Nikon, Tokyo, Japan). The microinjected embryos were examined, dead ones were removed, and the remaining embryos were used for analysis and breeding.

### 2.4. Identification of ins/del and Frameshift Mutations in SLR and GHR

To confirm the insertion/deletion (ins/del) and frameshift mutations generated in *SLR* and *GHR* by the CRISPR/Cas9 system, a heteroduplex mobility assay (HMA) was conducted as described elsewhere [16,18,19]. Briefly, genomic DNA was extracted from 4-day-old embryos or the caudal fins of adult fish, and amplified by PCR (initiated at 98 °C for 30 s, followed by 30 cycles of 98 °C for 20 s, 57 °C for 1 min, 68 °C for 30 s, and 72 °C for 10 min) using the KAPA *Taq* polymerase (Roche, Basel, Switzerland) and the following appropriate primers (at a final concentration of 0.5 µM each): F, 5′-GATAAGCTTGTAAGGTAAATATTGAGG-3′ and R, 5′-TCTCATTGCTCTCAAACAAATC-3′ for *SLR*; and F, 5′-GTTCAGTTTCCTTGTGTCTTATATTTTCTGTAAAGGTTAAG-3′ and R, 5′-GGAACGCTTTAAAAATAGATCACATGACCGTAG-3′ for *GHR*. The amplification products were then electrophorized on a 12% polyacrylamide gel.

G_0_ adults that were confirmed to carry ins/del mutations in the caudal fin were mated with WT medaka (HNI) to obtain heterozygous F_1_ fish with the ins/del mutations. To confirm the transmission of the frameshift mutations to the F_1_ generation, HMA and sequencing were performed using a part of the caudal fin from F_1_ adult fish, as described above. After males and females with identical heterozygous frameshift mutations were obtained, the F_1_ fish were mated to generate the F_2_ generation. If males and females with identical frameshift mutations were not obtained, the F_1_ fish were backcrossed with WT fish.

### 2.5. Analysis of Viability

The heterozygous F_1_ males and females carrying identical frameshift mutations were mated with each other and the fertilized F_2_ eggs were collected. The littermates with unknown genotypes were bred in the same tanks en masse (one or more tanks per batch, depending on the number of individuals) and were genotyped using whole larvae or the caudal fin of adults, as described above. The expected ratio of WT, heterozygote, and homozygote fish in the F_2_ generation was 1:2:1 at any stage if the viability did not depend on the genotype.

### 2.6. Analysis of Body Color and Body Length

The body color and body size in the F_2_ generation were examined among genotypes by counting the density of chromatophores and measuring the distance between the snout and the tip of the caudal fin, respectively. On the day of chromatophore counting, adult medaka (over 3 months old (F_2_)) were placed in a white tank for at least 30 min to induce melanophore aggregation. Subsequently, they were anesthetized on ice for 1–2 min, placed on a Petri dish, and the epidermis on the dorsal side of the trunk (near the base of the dorsal fin) was photographed under a stereoscopic microscope. The number of melanophores, xanthophores, and leucophores was counted manually in an area of 1 mm × 3 mm, using Image J^®^, to calculate the cell density (cells/mm^2^). Because xanthophores were difficult to distinguish in raw micrographs, the contrast was increased by adjusting the balance of the blue color. The color and size measurements of the F_2_ littermates were performed before the identification of the genotypes of the subjects via HMA or sequencing, as described above.

### 2.7. Reverse Transcription Polymerase Chain Reaction

To confirm the nonsense-mediated mRNA decay (NMD) caused by the mutations in *GHR*, a reverse transcription PCR (RT-PCR) was performed. Total RNA was extracted from the livers of three adult fish, each from the WT, homozygous for *ghr^+1^*, and homozygous for *ghr^−1^* groups, using ISOGENII (Nippon Gene). After treatment with deoxyribonuclease (RT Grade) for Heat Stop (Nippon Gene), the mRNA was reverse transcribed using a polyT primer (5′-ATTCTAGAGGCCGAGGCGGCCGACATGTTTTTTTTTTTTTTTTTVN-3′) and ReverTra Ace (Toyobo) to synthesize cDNA. All procedures were performed according to the manufacturers’ protocols. PCR was carried out as described above using the cDNA as a template and the following primers: F, 5′-GGTCTTCTGCTCATGCTCATGATGTC-3′ and R, 5′-CATTTCATGGTAGGAGGTTTCCCAGC-3′ for *SLR*; F, 5′-ATGGCGGCTGCGCTCAC-3′ and R, 5′-ATGCTGCTCAAAAGGTCAGGAATCAG-3′ for *GHR*; and F, 5′-ATGGATGATGACATTGCCGCACTG-3′ and R, 5′-TTAGAAGCATTTGCGGTGGACGATG-3′ for *beta-actin*. The expression of the transcripts was confirmed by electrophoresis on agarose gels with ethidium bromide staining. The number of PCR cycles was adjusted for each gene so that the WT band could be detected before reaching a plateau.

### 2.8. Statistics

The viability (ratio) of F_2_ offspring among genotypes (see Section 2.5) was analyzed using a chi-squared test with a null hypothesis of WT:hetero:homo = 1:2:1. The body length and chromatophore density between two or among three genotypes (see Section 2.6) were analyzed using Student’s *t*-test or one-way ANOVA, respectively. When there was a significant effect in the one-way ANOVA, the Tukey–Kramer test was additionally carried out to identify statistically significant differences among the levels (genotypes) of a parameter (body length or chromatophore density). Significant differences in the Tukey–Kramer test are indicated by *p* < 0.05. We did not correct the *p* value to reduce the type 1 error rate. The results are presented as the mean ± standard error (SE).

## 3. Results

### 3.1. Phylogenetic Reassessment of GHRa and GHRb in Teleosts

**Figure 2:**
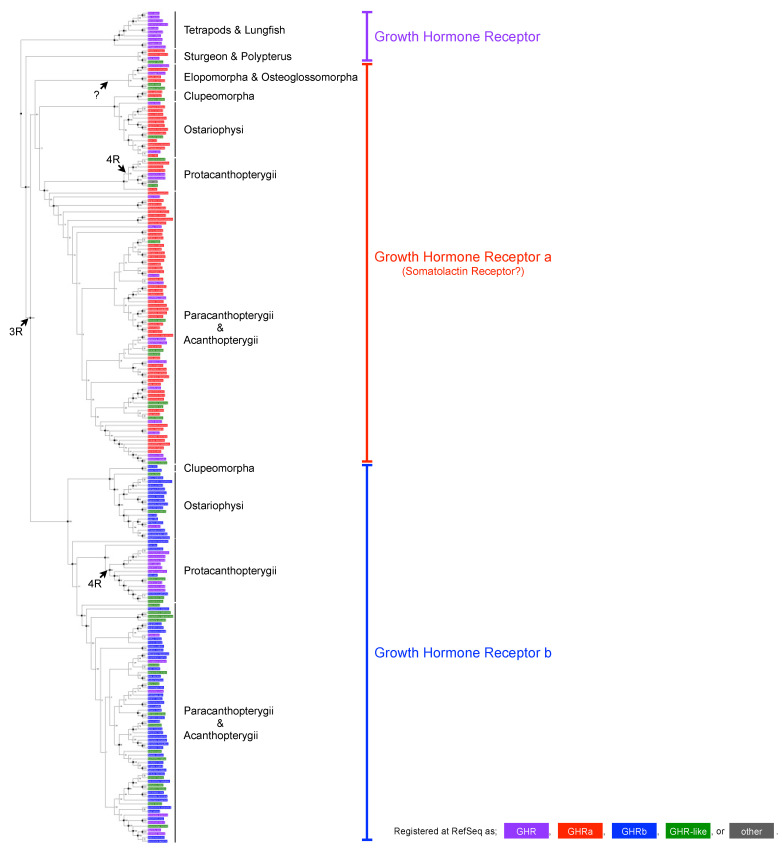
Cladogram of the GHR sequences registered at the RefSeq protein database. A total of 222 sequences (136 species) were used for phylogenetic tree reconstruction using the maximum-likelihood method. Sequences classified in the database as GHR, GHRa, GHRb, GHR-like, or other are colored in purple, red, blue, green, or gray, respectively. Bootstrap values are shown at each node, and nodes with a bootstrap value of 90% or more are labeled by black dots. Three groups (GHR of tetrapods, lungfish, sturgeon, and polypterus [purple]; GHRa [red] of teleosts; and GHRb [blue] of teleosts) are supported by bootstrap values of 100%. This figure revealed many problems in the nomenclature of GHRs.

The common ancestor of teleosts underwent a third-round (3R) genome duplication, and many teleosts have two copies of *GHR*, which are currently termed *GHRa* and *GHRb*, whereas other vertebrates (e.g., tetrapods, lungfish, sturgeon, and polypterus) have only one copy. An additional genome duplication (4R) occurred at the common ancestor of Salmoninae, resulting in four copies of *GHR*. This evolutionary scenario was largely (with the exception of Elopomorpha) supported by the phylogenetic reconstruction performed here, using a total of 222 sequences from 136 species (Figure 2). However, this analysis revealed serious problems in the current nomenclature, as exemplified below.

First, the GHRs of sturgeons and polypterus, which did not experience the 3R genome duplication, should simply be termed as “GHR” however, two of the four sequences were classified as “GHRa”, and one was defined as “GHR-like”. Second, two copies of GHR were found in Elopomorpha (but not in Osteoglossomorpha), both of which belonged to the GHRa clade (because of a lineage-specific *GHRa* duplication?); however, one was designated as “GHR-like” despite the fact that it must be a genuine GHR (GHRa). Third, there were many other “GHR-like” entries in the GHRa and GHRb clades, which confusingly and wrongly indicated that these sequences are similar to, but different from, GHR. Fourth, several other “GHR-like” entries in the database could not be included in the present phylogenetic reconstruction. Their phylogenetic positions clearly contradicted the traditional ones, indicating that they are not orthologous to GHR; rather, they seem to be genuine “GHR-like” receptors. Fifth, there were many “GHR” entries in the GHRa and GHRb clades, which obscured the paralogous relationships. Sixth, GHRa and GHRb (i.e., phylogenetically distinct receptors) were indistinguishably designated as “GHR” or “GHR-like” for many species (particularly *Salmoninae*, having four copies of GHR).

The sequences of medaka (*O. sakaizumii* or *O. latipes*) were also indistinguishably designated as “GHR” in the database, whereas the phylogenetic reconstruction clearly demonstrated that XP_011477148 (and NP_001098560) and NP_001116377 (and XP_023816119, XP_023816118, and XP_023816117) are GHRa and GHRb, respectively. The former and latter were originally designated as SLR (DQ002886) and GHR (DQ010539), respectively. Although we do agree with the GHRa/GHRb nomenclature, we followed the original SLR/GHR nomenclature in the present study.

### 3.2. Mutagenesis Efficiency

Before we finally decided on the target sequences for CRISPR/Cas9 (see Methods), a total of eight sequences were tested. Among the eight gRNAs (SLR5/6/7/8 and GHR5/6/7/8), we were not able to transcribe SLR6 and SLR7 sufficiently for unknown reasons; therefore, the mutagenesis efficacy was examined using the remaining six gRNAs (Table 1). Co-injection of each gRNA with the *Cas9* mRNA into 22–143 fertilized eggs yielded the 1-day viability, which ranged from 24.5% to 68.2% (Table 1). Hatched larvae injected with SLR8 or GHR6 were confirmed to have ins/del mutations in 35.7% or 25.0% of cases, respectively (Table 1), whereas SLR5, GHR5, GHR7, and GHR8 injections did not trigger any mutagenesis activity. Therefore, SLR8 and GHR6 were used thereafter as gRNAs for the *SLR* and *GHR* genes, respectively.

Using the genomic sequence of the HNI strain (version2.2.4; http://viewer.shigen.info/medaka/download.php, accessed on 25 March 2023), we manually verified that no genomic sequence other than the actual targets on chromosome 9 or 12 was identical to the 18 nucleotides adjacent to the PAM of SLR8 or GHR6 (i.e., TCCTGCGCCCAAAATCAA or CAGGAGCCTTGTGTTTGG). We also surveyed a genomic sequence using the Pattern Match program at the NBRP medaka (http://viewer.shigen.info/medakavw/crisprtool/, accessed on 25 March 2023); however, this tool is currently available only for the Hd-rR strain, *O. latipes*. When up to 2 mismatches were allowed in the 21-base target sequences, the 0 or 3 sequences could be potential off-targets for SLR8 or GHR6, respectively (Table 2). One of them was located about 320 kb downstream of the *GHR* locus, which is part of an intron of the *PDZ and pleckstrin homology domains 1* gene.

Identical/similar sequences could exist in the HNI genome, and these observations cannot perfectly exclude a possible off-target effect; however, the risk of the simultaneous induction of frameshift mutations at multiple loci (see Table 3) and their co-inheritance by F_1_ and F_2_ siblings should not be high (with the exception of the tightly linked off-target). We also assumed that an intronic ins/del mutation would hardly affect the phenotype. Hence, we decided to use the SLR8 or GHR6 gRNAs for mutagenesis, rather than designing/testing additional gRNAs.

The mixture of two gRNAs and the *Cas9* mRNA was microinjected into a total of 679 fertilized WT eggs. The mortality of embryos, the incidence of deformed embryos, and the incidence of normal development were 52.6%, 12.8%, and 34.6%, respectively. The overall efficiency of mutagenesis in the G_0_ larvae was predicted to be obtained with the following probabilities: fish with a single ins/del mutation on *SLR* (26.8%); fish with a single ins/del mutation on *GHR* (16.1%); fish with double ins/del mutations on both *SLR* and *GHR* (8.9%) (see Table 1). Of the 679 microinjected G_0_ eggs, 41 finally matured; therefore, the predicted values became 11.0 for a single mutation on *SLR*, 6.6 for a single mutation on *GHR*, 3.7 for double mutations on both *SLR* and *GHR*, and 19.8 for no mutations. The actual values as a result of HMAs were 8, 0, 7, and 26, respectively, which were significantly different from the predicted ones (*p* = 0.006, chi-squared test), albeit for an unknown reason.

A total of 9 out of the 15 G_0_ fish (numbered #1 to #15) that were confirmed to have ins/del mutations in the caudal fin were mated with WT fish or with each other, and 16 F_1_ larvae were collected from each cross. HMAs of the larvae revealed that 13 mutation types had been passed from 6 (or 7) G_0_ fish (#1, #2, #4 and/or #7, #5, #6, and #8) to the F_1_ generation, many of which were shown to be frameshift mutations by sequencing (Table 3).

Among them, the #1, #6, and #8 males with good growth conditions were preferentially selected, and 99, 128, and 176 F_1_ adult fish were obtained, respectively. A total of 12 and 4 mutation types were found, including 8 and 3 frameshift mutations in the *SLR* and *GHR* genes, respectively (Table 3). F_1_ fish with identical frameshift mutations in *SLR* were mated with each other to obtain the F_2_ generation. Regarding *GHR*, because the number of F_1_ adult fish with identical frameshift mutations was not sufficient for mating, they were backcrossed with WT fish to increase the number of heterozygotes.

### 3.3. Confirmation of Frameshift Mutations in slr and ghr Mutants

**Figure 3:**
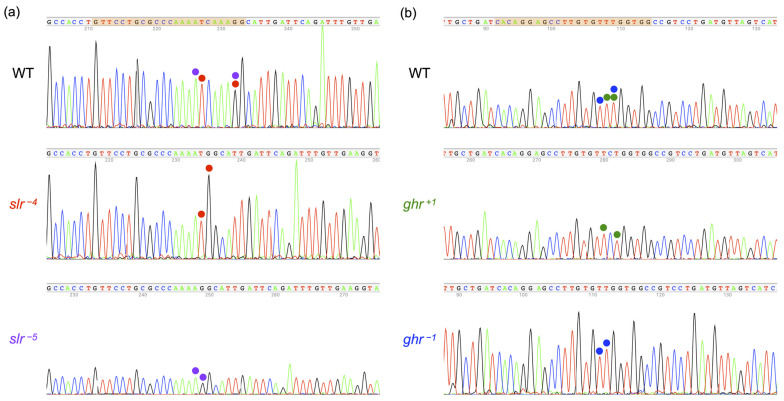
Electropherograms of target sequences and frameshift mutations in *SLR* and *GHR*. The target sequences (highlighted in orange) for the wild-type (WT) fish are shown at the top for *SLR* (**a**) and *GHR* (**b**). Corresponding nucleotides in the WT and mutant fish are marked by colored circles. Four bases (CAAA) or five bases (TCAAA) are deleted in *slr^−4^* and *slr^−5^*, respectively. One base (C or T) is inserted or deleted in *ghr^+1^* or *ghr^−1^*, respectively.

The successful introduction of frameshift mutations into *slr* and *ghr* mutants was confirmed by electropherograms of the target sequences: a 4- or 5-base deletion (CAAA or TCAAA) in *slr^−4^* or *slr^−5^* homozygotes, respectively (Figure 3a), and a 1-base insertion or deletion of C or T in *ghr^+1^* or *ghr^−1^* homozygotes, respectively (Figure 3b).

**Figure 4:**
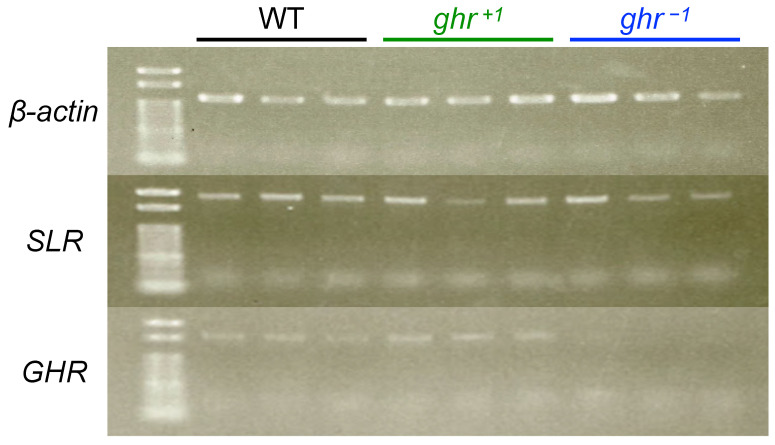
PT-PCR for three adults, each among WT, *ghr^+1^*, and *ghr^−1^* fish. The upper lanes, middle lanes, and bottom lanes show the expression of *beta-actin* (22 cycles), *SLR* (28 cycles), and *GHR* (28 cycles), respectively. The transcripts of *GHR* were not detected in all three *ghr^−1^* individuals, whereas these bands were observed in all WT and *ghr^+1^* individuals. Bands from RT-PCR products for *beta-actin* and *SLR* were detected in all individuals from all strains.

The frameshift mutations (premature stop codons) in *GHR* were also checked by NMD analysis. RT-PCR was performed using the total RNA from three adult fish livers, each among WT fish, *ghr^+1^* homozygotes, and *ghr^−1^* homozygotes; the products were electrophoresed on an agarose gel. The expression of *beta-actin* and *SLR* was detected in each individual from all strains (Figure 4). The expression of *GHR* was detected in WT and *ghr^+1^* individuals, whereas the apparent NMD could be detected in three *ghr^−1^* individuals, suggesting that the frameshift mutation successfully induced a premature stop codon in *GHR*, at least in *ghr^−1^* fish.

It was not possible to confirm NMD in *slr^−4^* or *slr^−5^* homozygotes because of death before maturity (see Section 3.4). The only adults obtained were four *slr^−5^* homozygotes, which were used in the body-color analysis (see Section 3.5) and were not obtained again in later crossings. Experiments using whole larvae might have solved this problem; however, the frameshift mutations do not always induce NMD (e.g., *ghr^+1^*) and, thus, we did not perform this experiment.

### 3.4. Viability and Body Size of Full Siblings Obtained by Crossing Heterozygous Mutants

The genotype ratio for WT, heterozygous, and homozygous fish among the siblings obtained between heterozygotes is expected to be 1:2:1. For *slr^−4^* siblings, the ratio was 75:114:0 in the adult stage (a sum of six independent crosses), and no homozygotes were detected, which was significantly different from the expected value of 47.25:94.5:47.25 (*p* < 0.001, chi-squared test; Table 4). This result clearly demonstrated that the *slr^−4^* mutation recessively suppresses the adult stage in medaka. For *slr^−5^* siblings, the ratio (a sum of three independent crosses) was 18:33:4, which was also different from the expected ratio of 13.75:27.5:13.75 (*p* = 0.009). We could not obtain fertilized eggs from the *slr^−5^* homozygotes or even *slr^−5^* heterozygotes, and this mutation was unfortunately lost. The frozen sperm of *slr^−4^* heterozygotes is available at the NBRP medaka (MT1356), together with those of missense mutants of *slr^C100F^* (MT1081) and *slr^F55I^* (MT1082).

For *ghr^+1^* and *ghr^−1^* siblings, the genotype ratios of 12:24:9 and 32:68:28 observed in adult fish (the sum of two or seven independent crosses, respectively) were not different from the expected values of 11.25:22.5:11.25 and 32:64:32, respectively (*p* = 0.741 or 0.687, respectively, chi-squared test; Table 4). The frozen sperm of *ghr^−1^* homozygotes is available at the NBRP medaka (MT1354), together with those of missense mutants of *ghr^P161S^* (MT1078), *ghr^V166L^* (MT1079), and *ghr^W103R^* (MT1080).

**Figure 5:**
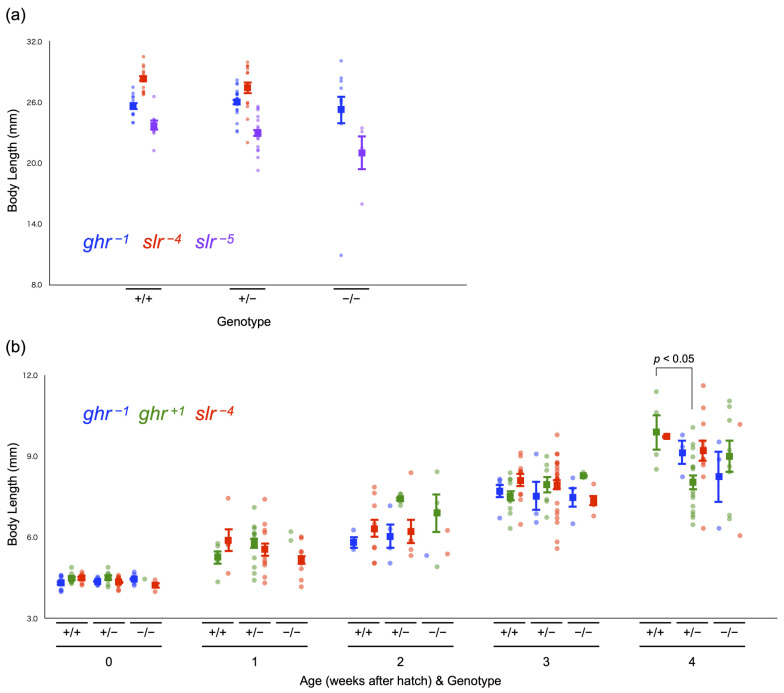
Body length at the adult and larval stages. (**a**) Body length of adult fish among genotypes in *ghr^−1^* (blue dots), *slr^−4^* (red dots), and *slr^−5^* (purple dots) individuals. (**b**) Transition of body length among genotypes during the larval stage in *ghr^−1^* (blue dots), *ghr^+1^* (green dots), and *slr^−4^* (red dots) individuals. Genotypes are indicated as +/+ (WT fish), +/− (heterozygotes), and −/− (homozygotes). Each dot represents the body length of each individual, and a closed box with bars indicates the mean and standard error.

We measured the body length of some of these adults (Figure 5a). Among *ghr^−1^* fish, the average body length of 5-month-old adult fish was similar among genotypes, as follows: WT: 25.6 ± 0.4 cm (*n* = 11), heterozygotes: 26.0 ± 0.3 cm (*n* = 23), and homozygotes: 25.3 ± 1.4 cm (*n* = 12) (mean ± SE; *p* = 0.736, one-way ANOVA). Although there were no homozygotes for *slr^−4^*, no difference in body length was observed between 4–11-month-old WT fish (28.3 ± 0.3 cm, *n* = 13) and heterozygotes (27.5 ± 0.6 cm, *n* = 14) (*p* = 0.249, Student’s *t*-test). For *slr^−5^* (7-month-old fish), no significant difference was detected among genotypes, as follows: WT: 23.7 ± 0.5 cm (*n* = 8), heterozygotes: 23.0 ± 0.4 cm (*n* = 18), and homozygotes: 21.0 ± 1.7 cm (*n* = 4) (*p* = 0.097, one-way ANOVA); however, there seemed to be a tendency for homozygotes to be smaller than WT and heterozygote fish.

We also examined the genotype ratio at the larval stage (0–4 weeks post-hatching (wph)) among *ghr^−1^*, *ghr^+1^*, and *slr^−4^* individuals. The ratios (a sum of 1–4 batches) fluctuated because of the small number of larvae included in the analysis (6–47 individuals per week per strain), which might accidentally have caused a significantly biased ratio at 3 wph in *ghr^+1^* fish (*p* = 0.035, chi-squared test; Table 5). We noted that the percentages of *slr^−4^* homozygotes at 2, 3, and 4 wph were consistently lower (by ~10%) than the expected value (25%), with one of these values (that obtained at 2 wph) being statistically significant (*p* = 0.027). This result indicates that *slr^−4^* homozygous larvae are less viable than the full siblings of other genotypes, and this resulted in the absence of homozygotes in the adult stage.

We measured the body length of all larvae before the genotyping analysis (Figure 5b). For *ghr^+1^* siblings, heterozygotes (8.0 ± 0.3 cm, *n* = 16) had a significantly shorter body length than WT fish (9.9 ± 0.7 cm, *n* = 4) at 4 wph (*p* = 0.036, one-way ANOVA and Tukey–Kramer test), whereas there was no difference among genotypes at other weeks of age (all *p* > 0.05, Student’s *t*-test or one-way ANOVA). Because we did not correct the *p* values to reduce the type 1 error rate, the above difference that was surprisingly detected between WT fish and heterozygotes could be an accidental event. For *ghr^−1^* and *slr^−4^*, no difference in body length was observed at any wph (all *p* > 0.05, one-way ANOVA). These results do not statistically support the contention that the death of *slr^−4^* homozygous larvae is caused by growth retardation, although there might be a trend toward this effect (e.g., at 1 and 3 wph).

### 3.5. Body Color of Full Siblings Obtained by Crossing Heterozygous Mutant Fish

**Figure 6:**
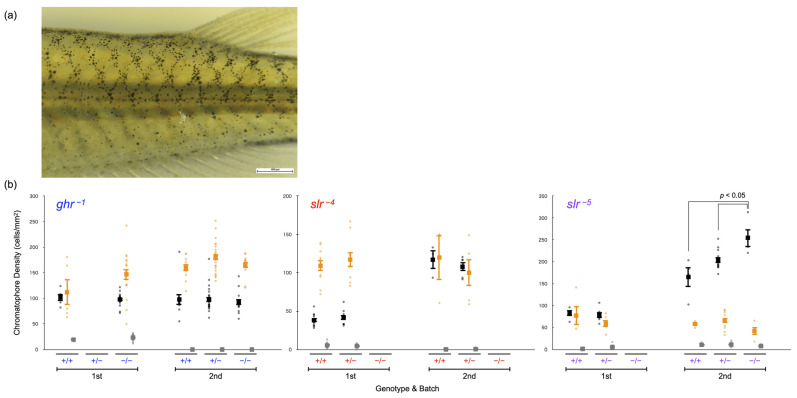
Comparison of body color. (**a**) Example of the images used for counting chromatophores. (**b**) Chromatophore densities in adult medaka. The numbers of melanophores (black dots), xanthophores (orange dots), and leucophores (gray dots) in *ghr^−1^*, *slr^−4^*, and *slr^−5^* adult fish examined twice in two different batches are shown. Genotypes are indicated as +/+ (WT fish), +/− (heterozygotes), and −/− (homozygotes). A dot represents the chromatophore density of each individual and a closed box with bars indicates the mean and standard error.

Lastly, we evaluated the body color (Figure 6), supposing that the density of leucophores and xanthophores in the *slr* mutants should, respectively, be increased and decreased if SLR functions as the receptor for SLα. The density of chromatophores in adult fish was examined twice for each mutation (*ghr^−1^*, *slr^−4^*, and *slr^−5^*) using independent batches. The results (body color) were not necessarily identical between the batches because the color of breeding tanks depends on accidental factors (e.g., algal growth) and medaka adapt their body color to the tank color. Therefore, the comparison among genotypes was performed only within (and not between) each batch.

Regarding the *slr^−4^* mutation, for which no homozygotes were obtained, there was no significant difference between WT fish (*n* = 10 or 3) and heterozygotes (*n* = 9 or 5) for any of the chromatophores (*p* > 0.05, Student’s *t*-test). Furthermore, no significant difference was detected between WT fish (*n* = 8) and *slr^−5^* heterozygotes (*n* = 18) in the first batch (*p* > 0.05, Student’s *t*-test). In the second batch, the density of melanophores in *slr^−5^* homozygotes (*n* = 4, 254.9 ± 20.6 cells/mm^2^) was greater than that observed in WT fish (*n* = 12, 165.3 ± 21.9 cells/mm^2^) and heterozygotes (*n* = 4, 203.2 ± 6.7 cells/mm^2^) (*p* = 0.003, one-way ANOVA and Tukey–Kramer test); in turn, the differences in xanthophores or leucophores were not significant (*p* > 0.05).

We also examined the body color of the *ghr^−1^* mutant and found no differences in any of the chromatophores among the genotypes (first batch: *n* = 5 and 17 for WT and homozygotes, respectively; second batch: *n* = 11, 23, and 12 for WT, heterozygotes, and homozygotes, respectively) (*p* > 0.05, Student’s *t*-test or one-way ANOVA).

## 4. Discussion

### 4.1. Confirmation of Frameshift Mutations in ghr/slr Mutants

We impaired the receptor function of SLR and GHR in medaka fish using the CRISPR/Cas9 system and used sequencing to confirm the successful generation of mutants carrying frameshift mutations (Figure 3). The introduction of the frameshift mutations into *ghr^−1^* mutants was also double-checked using NMD (Figure 4). NMD is one of the eukaryotic mRNA quality control mechanisms that selectively degrade abnormal mRNA before translation when an immature stop codon occurs in the translation region [20]. Bands from transcripts obtained by RT-PCR, that cannot be confirmed or can be judged to be fainter than that obtained for the WT, imply that the mRNA is degraded after transcription, and it can be judged that NMD has occurred. Because the mRNA was degraded in *ghr^−1^* fish because of NMD, an adequate amount of cDNA was not synthesized by reverse transcription, resulting in the lack of confirmation of *GHR* transcripts by RT-PCR.

Conversely, NMD does not always occur for mRNAs with immature stop codons [20]. The confirmation of the bands from transcripts implies that the transcribed RNA is retained without being degraded, and it can be judged that NMD has not occurred. The transcript products of *GHR* detected in all three *ghr^+1^* homozygotes seemed to be equivalent in amount to those detected in WT fish, indicating that NMD had not occurred in *ghr^+1^* homozygotes, although a functional GHR should not be transcribed from the mutated mRNA.

Regarding the *slr* mutations, we could not assess NMD because *slr^−4^* or *slr^−5^* homozygotes could never or seldom be obtained at the adult stage, respectively (Table 4). This severe mortality commonly shown by the *slr* mutants should support the successful introduction of frameshift mutations in the same gene, *SLR*. For instance, the establishment and analysis of multiple mutants could reduce the risk of misunderstanding the genotype–phenotype correlations caused by off-target effects.

### 4.2. Estimation of Medaka SLR Functions

SLR is supposed to be a receptor for SLα. Biallelic mutations in *SLR* in medaka decrease the melanophores in larval fish [21]; however, medaka homozygous for *slr^−5^* had more melanophores than WT fish. These results indicate that SLR participates in body color regulation to a certain extent (e.g., it darkens larvae but brightens adults). If SLR functions as a necessary and sufficient receptor for SLα, the SLR (receptor) mutants should have exhibited the same body color as the SLα (ligand) mutant, *ci*. However, the dramatic increase and decrease in leucophores and xanthophores, respectively, that have repeatedly been reported for the ligand mutant [2,3,8], were not at all reproduced in the receptor mutant (Figure 6).

In addition, the receptor mutations had a serious effect on vital activities before sexual maturity. Despite repeated attempts to preserve them, all individuals that were homozygous for *slr^−^^4^* died before maturity and we obtained only four *slr^−^^5^* homozygous adults that were unable to reproduce (Table 4). The results obtained for the receptor mutants were also incompatible with those of the ligand mutant, *ci*, which exhibited ordinary growth and viability [8].

The present results of a dissimilar body coloration in *slr^−5^* individuals compared with that of *ci* and the difficulty in growth (survival) commonly observed in the *slr^−5^* and *slr^−4^* mutants suggest one of the two following possibilities: (1) SLR functions as a receptor for SLα, but there is another factor (e.g., receptor) that compensates for the leucophore/xanthophore deficiency; or (2) SLR is a receptor for GH, rather than SLα. The phylogenetic reconstruction (Figure 2) showed that the medaka SLR was surely orthologous to the GHR of tetrapods. Moreover, we found several “GHR-like” sequences that did not belong to either the GHRa (SLR) or GHRb (GHR) clades. Based on the present results, we favor the second possibility described above, i.e., SLR is a receptor that is essential for normal growth and possibly binds to GH. The *slr* mutants might be unable to receive sufficient GH signals, resulting in low viability, although inhibition of larval growth could not clearly be demonstrated by measuring the body length (Figure 5b).

### 4.3. Estimation of Medaka GHR Functions

GHR mutations in humans, pigs, and mice have been shown to inhibit growth [22,23,24,25]; however, none of the medaka *ghr* mutants (but not the *slr* mutants) showed any specific phenotype regarding viability, body length, or body color, suggesting that GHR is unlikely to be essential for GH-mediated growth promotion or SLα-mediated body color regulation in medaka. When the effect of gene knockout is not reflected in the phenotype, the role of the gene cannot be elucidated; therefore, the reverse genetics method used in this study could not clarify the function of medaka GHR. The phylogeny (Figure 2) at least supported the contention that medaka GHR (and SLR) is orthologous to tetrapod GHR. Even if both medaka orthologs could function as receptors for GH, the present results indicate that the role of GHR (but not SLR) is dispensable for growth. We were interested in the phenotypes of SLR/GHR double mutants and intercrossed the double heterozygotes. However, the high mortality of the *slr* mutations did not allow the establishment of the double-mutant lines (similar to the single mutants; Table 4).

We hope that the function of GHR will be elucidated by methods other than reverse genetics or by reverse genetics in other fish species. In addition, it is possible that SLR and GHR complement each other’s functions. If so, SLR/GHR double-knockout lineages need to be established and their phenotypes must be examined in the future.

## 5. Conclusions

A phylogenetic tree was systematically reconstructed, and a GHRa/GHRb nomenclature, instead of the SLR/GHR nomenclature, was proposed based on the positions of the gene sequences. SLR and GHR mutants were successfully established in medaka, and *slr^−4^*, *slr^−5^*, *ghr^+1^*, and *ghr^−1^* were selected for phenotyping. The *slr* mutants exhibited premature lethality, indicating the inactivation of the GH signal at the receptor. In turn, the *ghr* mutations did not affect viability, body length, or body color. These results indicate that the medaka GHR paralogs would phylogenetically and functionally be GH receptors. The receptor for SLα remains an open question.

## Figures and Tables

**Table 1:** gRNA target sequences and mutagenesis efficiency for the *SLR* and *GHR* genes in G_0_ fish.

gRNA	Target Sequence (5′→3′)	Eggs Injected (N)	Survivors on the Day Following Injection (N)	Survivors on the Day Following Injection (%)	Normal Development (N)	Larvae Subjected to HMA (N)	Larvae Confirmed to Have a Band Shift (N)	Mutagenesis Efficiency (%)
SLR5	CCAGAGTCAGAGGGGCCGTGG	40	16	40.0	15	15	0	0
SLR6	TCTCATTTTTGGGACTGTTGG	―	―	―	―	―	―	―
SLR7	TTTGGGCGCAGGAACAGGTGG	―	―	―	―	―	―	―
**SLR8**	**TCCTGCGCCCAAAATCAAAGG**	**29**	**15**	**51.7**	**14**	**14**	**5**	**35.7**
GHR5	GATCAGCAAAGCCATCACTGG	143	35	24.5	11	11	0	0
**GHR6**	**CAGGAGCCTTGTGTTTGGTGG**	**127**	**48**	**37.8**	**17**	**16**	**4**	**25.0**
GHR7	GGTTCAGGAACAGGAGGCAGG	35	20	57.1	19	13	0	0
GHR8	TCCTGAACCTAAAATAAAAGG	22	15	68.2	15	12	0	0

SLR8 and GHR6, which were finally selected as gRNAs for the *SLR* and *GHR* genes, respectively, are highlighted in bold.

**Table 2:** Potential off-targets of the GHR6 gRNA in the Hd-rR genome.

gRNA	Potential Off-Target Sequence (5′→3′)	Chromosome
GHR6	CAGGAGCCTTGTGTTTGGTGG	12 (target)
	CAGGAG**A**CTTGTGTTTG**T**GGG	1
	CAGGAGCC**A**TGT**T**TTTGGGGG	12
	GAG**C**AGCCTTGTGTTTG**A**GGG	19

**Table 3:** Ins/del mutations in *SLR* and *GHR* inherited by the F_1_ generation from the G_0_ fish.

Target Gene	G_0_ Individual Number	Mutation Type	Mutation	Sequence (5′→3′)	Number of F_1_ Larvae with Mutations (%)	Number of F_1_ Adults with Mutations (%)
SLR	-	WT	-	GCCCAAAATCAAAGGCATTGATTCAGATTTGTTGAAG	-	-
	**1**	six-deletion	SLR-6 (1)	GCCCAAAATCA––––––TTGATTCAGATTTGTTGAAG	3 (18.8)	13 (13.1)
	2	one-deletion	SLR+3-4	GCCCAAAA–CAGAGGCATTGATTCAGATTTGTTGAAG	1 (6.3)	0 (0)
	4 × 7	nine-insertion	SLR+9	GCCCAAAAATCAATGAATCAAAGGCATTGATTCAGATTTGTTGAAG	2 (12.5)	0 (0)
	4 × 7, **6**	five-deletion	**SLR-5**	**GCCCAAAA–––––GGCATTGATTCAGATTTGTTGAAG**	4 (18.8, 6.3)	5 (3.9)
	4 × 7, **8**	six-deletion	SLR-6 (2)	GCCCAAA––––––GGCATTGATTCAGATTTGTTGAAG	2 (12.5)	3 (1.7)
	5, **6**	two-insertion	SLR+2 (1)	GCCCAAACAATCAAAGGCATTGATTCAGATTTGTTGAAG	1 (6.3)	1 (0.8)
	**6**	sixteen-insertion	SLR+17-1	GCCCAAAAGAGCCTGAAACGATGTCCAAAGGCATTGATTCAGATTTGTTGAAG	0 (0)	3 (2.4)
	**6**	four-deletion	**SLR-4**	**GCCCAAAAT––––GGCATTGATTCAGATTTGTTGAAG**	1 (6.3)	9 (7)
	**6**	three-deletion	SLR-3	GCCCAAAA–––AAGGCATTGATTCAGATTTGTTGAAG	0 (0)	3 (2.3)
	**6**	two-insertion	SLR+2 (2)	GCCCAAAATGCCAAAGGCATTGATTCAGATTTGTTGAAG	1 (6.3)	9 (7)
	**6**	two-deletion	SLR-2	GCCCAAAA––AAAGGCATTGATTCAGATTTGTTGAAG	0 (0)	2 (1.6)
	**8**	seven-deletion	SLR+6-13	GCCCAAAGGTCTT–––––––ATTCAGATTTGTTGAAG	0 (0)	1 (0.6)
	**8**	six-insertion	SLR+7-1	GCCCAAAAATGGGCCCAAAGGCATTGATTCAGATTTGTTGAAG	2 (12.5)	4 (2.3)
	**8**	one-insertion	SLR+1	GCCCAAAATTCAAAGGCATTGATTCAGATTTGTTGAAG	2 (12.5)	29 (16.5)
				Total	19	82
GHR	-	WT	-	GCCTTGTGTTTGGTGGCCATCCTGATGTTAGTCATCA	-	-
	**1**	one-insertion	**GHR+1**	**GCCTTGTGTTCTGGTGGCCATCCTGATGTTAGTCATCA**	1 (6.3)	4 (4)
	4 × 7, **8**	one-deletion	**GHR-1**	**GCCTTGTGTT–GGTGGCCATCCTGATGTTAGTCATCA**	3 (12.5, 6.3)	5 (2.8)
	**6**	six-insertion	GHR+6	GCCTTGTGTGGTGCCTTGGTGGCCATCCTGATGTTAGTCATCA	1 (6.3)	7 (5.5)
	**8**	two-deletion	GHR-2	GCCTTGTGT––GGTGGCCATCCTGATGTTAGTCATCA	0 (0)	2 (1.1)
				Total	5	18

The wild-type (WT) sequence is shown at the top for each gene. Three G_0_ individuals, #1, #6, and #8, who were subjected to pass the mutations to F_1_ adult fish are highlighted in bold. Inserted or deleted nucleotides are shown as red uppercase letters or red hyphens, respectively. The mutants were named based on the number of ins/del mutations in *SLR* and *GHR* (e.g., *slr^−5^* means that five nucleotides were deleted from WT *SLR*). Four mutations, *slr^−5^*, *slr^−4^*, *ghr^+1^*, and *ghr^−1^*, were subjected to phenotyping and are highlighted in bold.

**Table 4:** Viability of *slr^−4^*, *slr^−5^*, *ghr^+1^*, and *ghr^−1^* among the genotypes of F_2_ adult fish.

Strain	Genotypes		
	*+/+*	*+/−*	*−/−*
*slr^–4^*	75	114	0
	0.000		
*slr^–5^*	18	33	4
	0.009		
*ghr^+1^*	12	24	9
	0.741		
*ghr^–1^*	32	68	28
	0.687		

Genotypes are indicated as *+/+* (WT fish), *+/−* (heterozygotes), and *−/−* (homozygotes).

**Table 5:** Viability of *ghr^+1^*, *ghr^−1^*, and *slr^−4^* F_2_ larval fish according to genotype.

Strain	Age (weeks) and Genotype														
	0			1			2			3			4		
	+/+	+/−	−/−	+/+	+/−	−/−	+/+	+/−	−/−	+/+	+/−	−/−	+/+	+/−	−/−
*ghr^+1^*	7	7	1	5	12	2	0	4	4	10	7	3	4	16	8
	0.088			0.323			0.135			0.035			0.424		
*ghr^–1^*	7	7	7	0	0	0	3	4	1	5	4	4	0	3	3
	0.311			―			0.607			0.354			0.223		
*slr^–4^*	10	18	9	8	18	15	9	6	2	15	27	5	4	11	2
	0.960			0.223			0.027			0.071			0.379		

Upper line: number of surviving individuals. Lower line: *p* value according to the chi-squared test. Genotypes are indicated as +/+ (WT fish), +/− (heterozygotes), and −/− (homozygotes).

## Data Availability

Data will be made available on request.

## Supplementary Materials

The following supporting information can be downloaded at: https://www.mdpi.com/article/10.3390/genes14040796/s1, Table S1: A list of sequences used for the phylogenetic reconstruction.
